# The Impact of Reimbursement Practices on the Pharmaceutical Market for Off-Patent Medicines in Slovakia

**DOI:** 10.3389/fphar.2021.795002

**Published:** 2021-12-13

**Authors:** Tomas Tesar, Peter Golias, Lucia Masarykova, Paweł Kawalec, András Inotai

**Affiliations:** ^1^ Department of Organization and Management of Pharmacy, Faculty of Pharmacy, Comenius University, Bratislava, Slovakia; ^2^ Institute for Economic and Social Reforms, Bratislava, Slovakia; ^3^ Faculty of Health Sciences, Institute of Public Health, Jagiellonian University Medical College, Krakow, Poland; ^4^ Syreon Research Institute, Budapest, Hungary; ^5^ Center of Health Technology Assessment, Semmelweis University, Budapest, Hungary

**Keywords:** off-patent medicines, reimbursement, pharmaceutical market, V4, Slovakia

## Abstract

**Background:** The aim of this study was to investigate the impact of selected legislative initiatives and their implementation for off-patent medicinal products in Slovakia compared with the rest of the Visegrád Group (V4 countries).

**Methods:** We analyzed the development of applications for the reimbursement of generic and biosimilar drugs. Particular emphasis was placed on a) the availability and penetration of biosimilars from 2006 to 2020 in Slovakia, b) a comparative analysis of biosimilars in V4 countries based on the national reimbursement lists of medicinal products for August 2021. Data relating to the sales of generic and biosimilar medicines in Czechia, Hungary, Poland, and Slovakia were based on the IQVIA MIDAS MAT July 2021.

**Results:** The number of applications for the reimbursement of generic drugs decreased from 296 in 2016 to 165 in 2020. In financial terms, the sales of generic medicines in Slovakia increased from 21.7% in 2015 to 22.3% in 2020. Over the same period, the sales of generic drugs in Poland fell from 40.4% in 2015 to 35.0% in 2020, from 26.2 to 22.1% in Hungary, and from 29.6 to 20.4% in Czechia. When considering the 66 biosimilars registered by the European Medicines Agency 38 drugs (58%) were available on the Slovak market as of August 1, 2021; this compared to 32 drugs (48%) in Poland, 38 drugs (58%) in Hungary, and 40 drugs (61%) in Czechia. In financial terms, the sales of biosimilars in Slovakia increased from 0.94% in 2015 to 2.00% in 2020. Over the same period, the sales of biosimilars in Poland increased from 0.59% in 2015 to 1.29% in 2020, from 0.72 to 2.23% in Hungary, and from 0.76 to 2.15% in Czechia.

**Conclusion:** To intensify the use of generic and biosimilar medicines, we suggest the comprehensive re-evaluation of combinations of the three-threshold entry, the amount of mandatory price reductions, and external reference pricing requirements (as the average of the three lowest prices among the official prices of a medicinal product in other Member States) for generic and biosimilar drugs. We also suggest cancellation of the exception from the fixed co-payment of the insured.

## Introduction

The aim of this study was to investigate the impact of selected legislative initiatives and their implementation for off-patent medicinal products in Slovakia, as compared with the rest of the countries in the Visegrád (V4) Group, which qualify as emerging markets (MSCI, 2021). The Visegrád Group is recognized as a cultural and political alliance of Czechia, Hungary, Poland, and Slovakia.

The basic concept of the Slovak health care system includes mandatory public insurance and a general benefits package for citizens (Smatana et al., 2016). The Slovak reimbursement and pricing system for medicinal products, including generics and biosimilars, has been described in the scientific literature (Tesar et al., 2019). In Slovakia, the European reference price for a medicine is defined by Act No. 363/2011 as “the average of the three lowest prices among the official prices of a medicinal product in other Member States” (Ministry of Health, 2011). From 2019, mandatory deductions based on the legislation have required that the first generic medicinal product arriving on the Slovak market has to show a 45% initial price reduction when compared to the original medicinal product; the second generic additional 10% price reduction compared to the first, and for the third generic, an additional 5% price reduction compared to the second. Furthermore, the first biosimilar arriving on the Slovak market has to show a 25% initial price reduction compared to the reference medicinal product; the second biosimilar an additional 5% price reduction compared to the first, and the third biosimilar an additional 5% price reduction compared to the second.

Mandatory deductions are thus applied from a relatively low price that is determined by an international reference; this may reduce the attractiveness of the Slovak market from the point-of-view of manufacturers responsible for both generic and biosimilar medicines. Slovakia is the only country in which the three-threshold entry (the obligation to reduce the price for the first three off-patent medicines when entering the Slovak market) is used in combination with external referencing and is one of the countries with the highest mandatory price reductions and the strictest external referencing (Golias, 2021). Therefore, it is important to regularly evaluate the impact of strict regulation on the entry of new off-patent medicines onto the market. There is a clear lack of current information relating to the impact of the above-described reimbursement practices on the pharmaceutical market for off-patent medicines in Slovakia.

In 2020, the COVID-19 pandemic had a significant effect on budget management in the Slovak Republic; this led to a 4.8% year-on-year reduction in the Slovak economy. According to the deficit and debt report, the general government budget management for 2020, according to European System of Accounts (ESA) 2010 methodology, reached a deficit of 6.16% of the gross domestic product (GDP). By the end of 2020, the consolidated general government debt, according to ESA 2010 methodology, exceeded 55 billion euros, thus representing 60.6% of the GDP. Year-on-year, the debt in relation to the GDP increased by almost 10 billion euros to 12.4%. This increase was predominantly due to the state budget deficit (amounting to 7.8 billion euros) according to the Supreme Audit Office of the Slovak Republic (2021).

The Supreme Audit Office (SAO) of the Slovak Republic drew attention to several key risks for austerity with relation to the medicines policy for 2021 and proposed several methods to support the financial sustainability of the medicines policy by making savings in the financial resources gained from public health insurance (Supreme Audit Office of the Slovak Republic, 2020).

Previous research has already highlighted that Slovakia has limited financial resources to reimburse medicinal products and that generic and biosimilar medicines can help to ensure financial sustainability of the pharmaceutical budget (Tesar et al., 2019). A previous study has also shown that Slovakia is one of the states within the European Union that has significant potential to improve health outcomes without increasing financial resources (Kuenzel and Solanic, 2018). Therefore, it is important that we implement reimbursement practices for off-patent medicines that maximize the social benefits of generic and biosimilar medicines for patients in Slovakia (Tesar et al., 2020).

In general, only medicines included in positive drug lists (PDLs) can be reimbursed by public health insurance (Löblová et al., 2019a). The consequences of not applying for pricing and reimbursement status by the marketing authorisation holder (as a result, medicinal products are not included in the PDL) are that the medicines are not sold and reimbursed from public health insurance funds in Slovakia; therefore, Slovak patients frequently have no access to these medicinal products.

If a medicinal product is not included in a PDL, but the marketing authorisation holder applied for pricing status only (the regulation of its ex-factory price applying the external reference pricing methodology by the Slovak Ministry of Health), the medicine can be sold and paid for out-of-pocket.

However, the Slovak “extraordinary reimbursement regime” (ERR) allows health insurance funds to allocate reimbursement to individual patients for medicinal products that are not on the country’s PDL (Löblová et al., 2019b), which brings limited access to Slovak patients for medicinal products in specific Anatomical Therapeutic Chemical classification system groups.

A substantial decrease in the number of applications for the reimbursement of generic drugs, and the low penetration of several biosimilar drugs, are connected to needless financial expenditures; therefore, valid data about the impact of reimbursement practices on the pharmaceutical market for off-patent medicines, as discussed in this paper, are needed for appropriate decision making concerning the national drug policy in Slovakia.

## Materials and Methods

We reviewed legislation that was relevant to our study. First, we reviewed Act No. 577/2004; this Act concerns the scope of health care that is paid for by public health insurance and by reimbursements for services related to the provision of health care (Ministry of Health, 2004). We also reviewed Act No. 363/2011; this Act relates to the scope and conditions of payments for medicines, medical devices, and dietetic foods, from public health insurance (Ministry of Health, 2011). Next, we analyzed the development of applications for the reimbursement of generic and biosimilar drugs. Such applications are officially issued on a website concerning the reimbursement of medicinal products and reported by the Slovak Ministry of Health (Ministry of Health, 2021a). The publicly available European Medicines Agency (EMA) website (EMA, 2021) was used to deliver the list of biosimilars for which the EMA granted marketing authorization (MA).

In this study, we focused on specific factors: a) the availability and penetration of biosimilars from January 2006 to December 2020 in Slovakia, b) a comparative analysis of the availability of biosimilars in the Visegrád Group based on the national reimbursement lists of medicinal products that were valid in the V4 countries in August 2021 [Ministry of Health, (2021b); Hungarian National Health Insurance Fund Administration, (2021); State Institute for Drug Control, (2021); Polish Ministry of Health, (2021)]. The State Institute for Drug Control and the National Health Information Centre were used as data sources for the sales of biosimilars and used to investigate the availability and penetration of biosimilars. The State Institute for Drug Control represents the Slovak National Authority in the area of human pharmacy (SIDC, 2021). Access to the database is only by request and based on the payment of a fee (MCR, 2021). The National Health Information Centre is responsible for delivering data relating to health statistics (NHIC, 2021); this database is free of charge and is publicly available.

The sales (percentage (%) of defined daily doses and % of financial terms) of generic and biosimilar medicines in Czechia, Hungary, Poland, and Slovakia were described based on the IQVIA MIDAS MAT 05/2021, July 2021 (IQVIA, 2021a) for 2015 and 2020. According to the World Health Organization (WHO), the defined daily dose (DDD) is the assumed average maintenance dose per day for a drug used for its main indication in adults (WHO, 2021). The IQVIA provides a range of data, including sales data for each product pack registered in the V4 markets; the data from database is available on request and based on a fee payment. (IQVIA, 2021b).

## Results

The price conditions for the reimbursement of generic and biosimilar drugs in Slovakia are summarized in Table 1.

**TABLE 1:** Development of price conditions for the reimbursement of generic and biosimilar drugs in Slovakia.

	Generics	Biosimilars
Act No. 577/2004 effective from November 1, 2004	No regulation	No regulation
Amendment effective from April 1, 2009	The first generic medicinal product arriving in the Slovak market has to bring a 20% initial price decrease compared to the original medicinal product	No regulation
Act No. 363/2011 effective from December 1, 2011	The first generic medicinal product arriving in the Slovak market has to bring a 30% initial price decrease compared to the original medicinal product	No regulation
Amendment effective from January 1, 2013	The first generic medicinal product arriving in the Slovak market has to bring a 35% initial price decrease compared to the original medicinal product	The first biosimilar arriving in the Slovak market has to bring a 20% initial price decrease compared to the References medicinal product
Amendment effective from od January 1, 2018	The first generic medicinal product arriving in the Slovak market has to bring a 45% initial price decrease compared to the original medicinal product, the second generic has to bring an additional 10% price decrease compared to the first, and the third generic has to bring an additional 5% price decrease compared to the second	The first biosimilar arriving in the Slovak market has to bring a 30% initial price decrease compared to the References medicinal product, the second biosimilar has to bring an additional 5% price decrease compared to the first, and the third biosimilar has to bring an additional 5% price decrease compared to the second
Amendment effective from January 1, 2019	The first generic medicinal product arriving in the Slovak market has to bring a 45% initial price decrease compared to the original medicinal product, the second generic has to bring an additional 10% price decrease compared to the first, and the third generic has to bring an additional 5% price decrease compared to the second	The first biosimilar arriving in the Slovak market has to bring a 25% initial price decrease compared to the References medicinal product, the second biosimilar has to bring an additional 5% price decrease compared to the first, and the third biosimilar has to bring an additional 5% price decrease compared to the second

**FIGURE 1:**
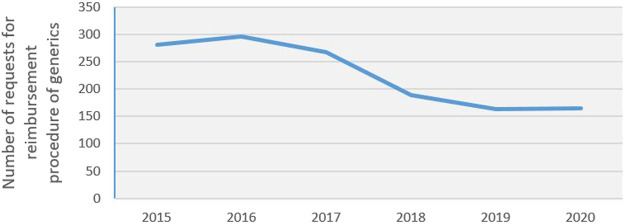
The number of applications for the reimbursement of generic drugs from 2015 to 2020.

**FIGURE 2:**
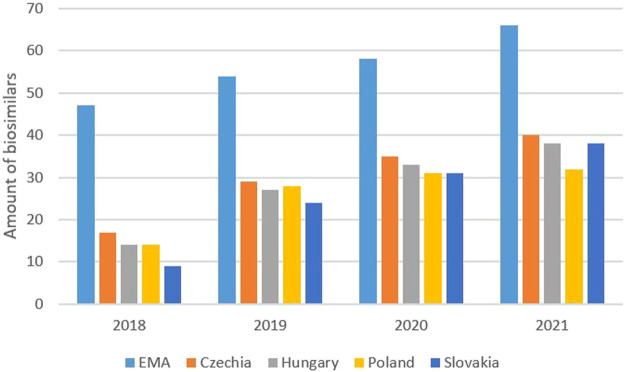
Availability of biosimilars for 2018, 2019, 2020 and August 2021.

In Slovakia, the number of applications for the reimbursement of generic drugs decreased from 296 in 2016 to 164 in 2019, representing a reduction of 45% over 3 years. There were 165 applications in 2020; see Figure 1 for more details. The detailed availability of biosimilars in the V4 group, as based on the reimbursement list from August 2021, is described in Table 2. Of the 66 biosimilars registered by the EMA, 38 drugs (58%) were available on the Slovak market as of August 1, 2021; this compared to 32 drugs (48%) in Poland, 38 drugs (58%) in Hungary, and 40 drugs (61%) in the Czech Republic. The development of availability of biosimilars for 2018, 2019, 2020, and August 2021 are shown in Figure 2. Of the 47 biosimilars registered by the EMA, 9 drugs (19%) were available on the Slovak market as of August 1, 2018; this compared to 14 drugs (30%) in Poland, 14 drugs (30%) in Hungary, and 17 drugs (36%) in the Czech Republic. Of the 54 biosimilars registered by the EMA, 24 drugs (44%) were available on the Slovak market as of August 1, 2019; this compared to 28 drugs (52%) in Poland, 27 drugs (50%) in Hungary, and 29 drugs (54%) in the Czech Republic. Of the 58 biosimilars registered by the EMA, 31 drugs (53%) were available on the Slovak market as of August 1, 2020; this compared to 31 drugs (53%) in Poland, 33 drugs (57%) in Hungary, and 35 drugs (60%) in the Czech Republic.

**TABLE 2:** Availability of biosimilars in V4 in August 2021.

Active substance (number of biosimilars received MA)	Number of available biosimilars			
	Czechia	Hungary	Poland	Slovakia
Adalimumab (8)	6	5	4	5
Bevacizumab (8)	5	3	2	5
Enoxaparin sodium (1)	1	0	0	0
Epoetin alfa (3)	1	1	1	1
Epoetin zeta (2)	0	1	0	0
Etanercept (3)	2	2	2	2
Filgrastim (7)	3	4	3	3
Follitropin alfa (2)	0	2	2	2
Infliximab (4)	4	3	4	3
Insulin aspart (2)	0	0	0	0
Insulin glargine (2)	2	1	1	2
Insulin lispro (1)	0	0	0	0
Pegfilgrastim (7)	5	4	4	5
Rituximab (6)	2	3	2	3
Somatropin (1)	1	1	1	0
Teriparatide (3)	2	2	0	2
Trastuzumab (6)	6	6	6	5
Total (66)	40	38	32	38

**FIGURE 3:**
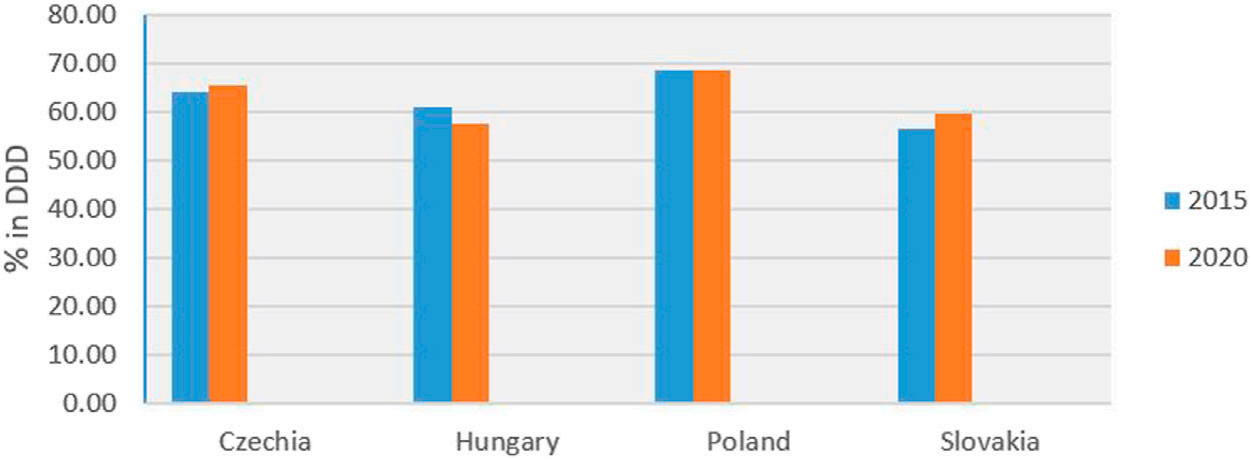
Consumption of generic medicines in % of DDD for 2015 and 2020.

**FIGURE 4:**
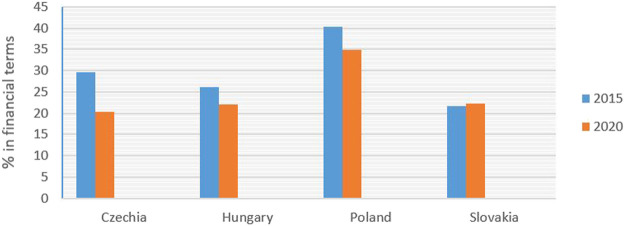
Consumption of generic medicines in % of financial terms for 2015 and 2020.

Analysis based on the DDD showed that sales of generic medicines in Slovakia increased from 56.66% in 2015 to 59.67% in 2020 (Figure 3). Over the same period, generic sales fell from 68.70% in 2015 to 68.44% in 2020 in Poland, from 61.10 to 57.70% in Hungary, and increased from 64.07 to 65.50% in Czechia. Financial analysis showed that sales of generic medicines in Slovakia increased from 21.65% in 2015 to 22.25% in 2020 (Figure 4). Over the same period, generic sales fell from 40.35% in 2015 to 34.97% in 2020 in Poland, from 26.20 to 22.08% in Hungary, and from 29.62 to 20.42% in Czechia.

**FIGURE 5:**
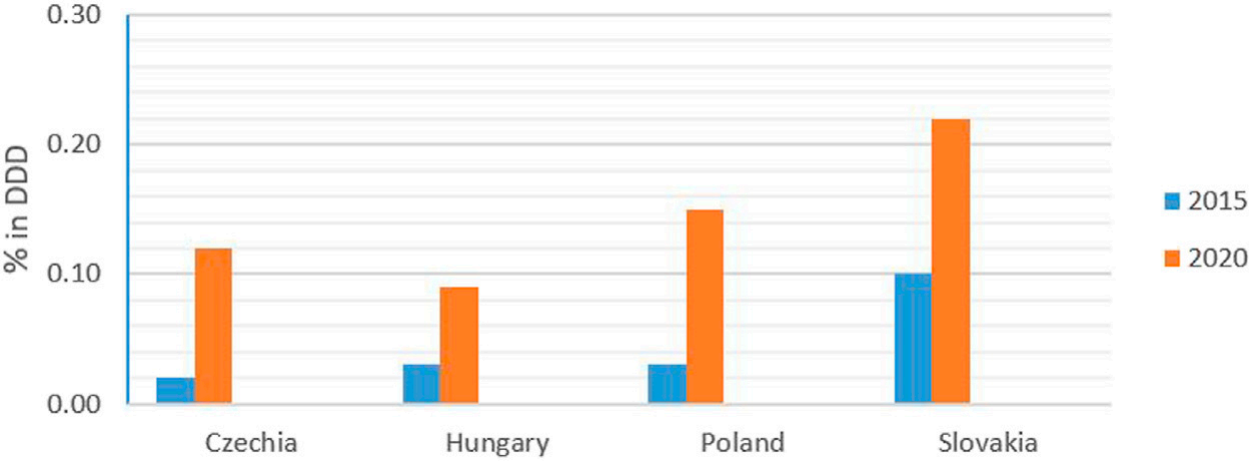
Consumption of biosimilars in % of DDD for 2015 and 2020.

**FIGURE 6:**
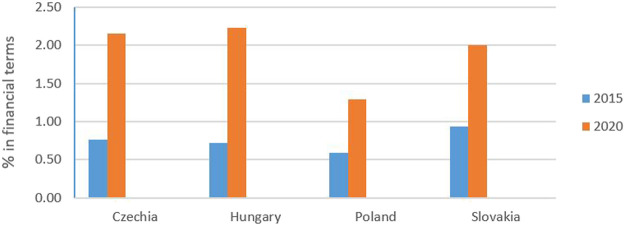
Consumption of biosimilars in % of financial terms for 2015 and 2020.

In terms of the DDD, sales of biosimilars in Slovakia increased from 0.10% in 2015 to 0.22% in 2020. Over the same period, sales of biosimilars increased from 0.03% in 2015 to 0.15% in 2020 in Poland, from 0.03 to 0.09% in Hungary, and from 0.02 to 0.12% in Czechia, as shown in Figure 5. Sales of biosimilars in Slovakia increased from 0.94% in 2015 to 2.00% in 2020. Over the same period, sales of biosimilars in Poland increased from 0.59% in 2015 to 1.29% in 2020, from 0.72 to 2.23% in Hungary, and from 0.76 to 2.15% in Czechia, as shown in Figure 6.

**FIGURE 7:**
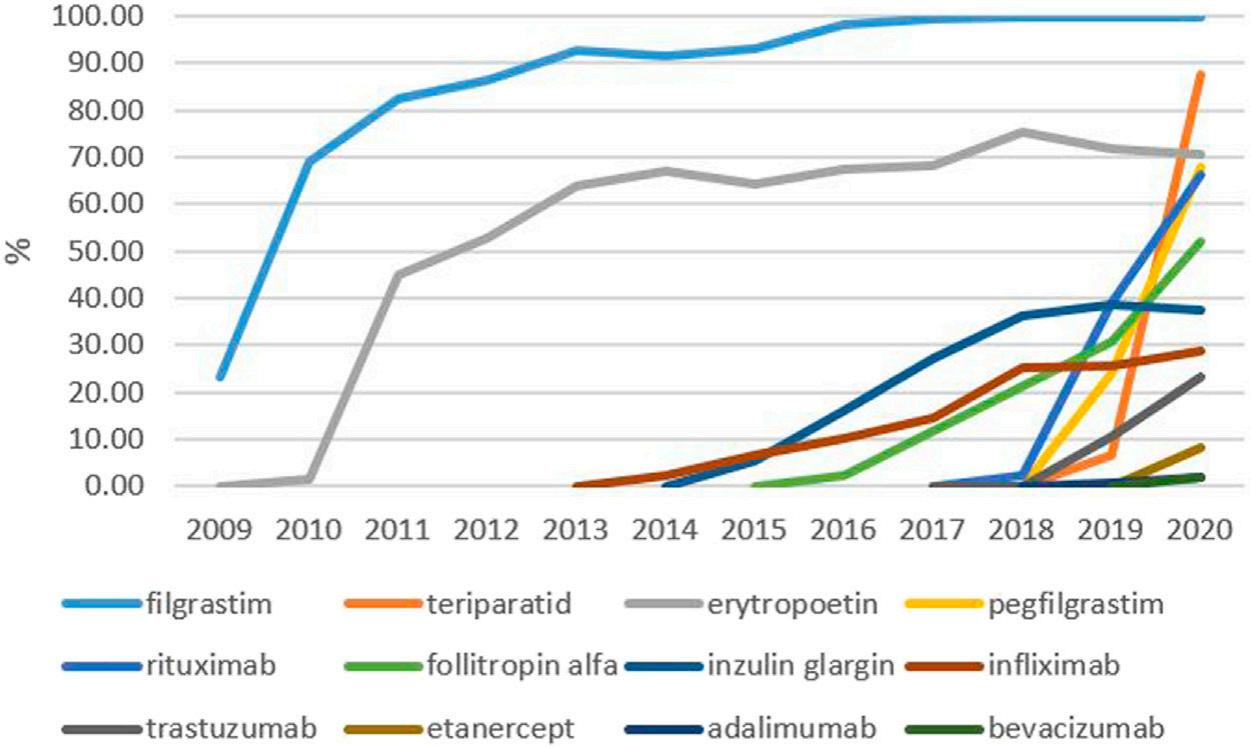
Biosimilar penetration in Slovakia (%).

Developments with regards to the penetration of biosimilars from 2006 to 2020 in Slovakia are shown in Figure 7.

Analysis of the development of biosimilar shares on the Slovak market according to specific molecules revealed a sharp increase for filgrastim and erythropoietin following their market entry in 2009–10. Biosimilars for filgrastim gradually occupied the entire market while shares of erythropoietin biosimilars have remained at approximately 70% for a long period of time now. Biosimilars for infliximab, insulin glargine, and follitropin alfa, arrived on the market later; shares for infliximab and insulin glargine remain at 30–40% but have reached 50% for follitropin alfa. Biosimilars for rituximab, trastuzumab, and adalimumab, entered the market at the end of 2018. Shares for rituximab rose rapidly to 66% in 2020. Shares for trastuzumab reached 23%, while those for adalimumab did not exceed 2%. Biosimilars for pegfilgrastim and etanercept were reimbursed from April and July 2019, respectively. Shares for pegfilgrastim biosimilars increased to 68% by the end of 2020; shares for etanercept reached 8%. At the end of 2019, the first biosimilar for teriparatide was reimbursed; the shares for this drug increased to almost 88% in 2020. In 2020, the first biosimilar for bevacizumab also came onto the market; this reached a market share of less than 2% by the end of the year. With regards to saving public resources, these low proportions of biosimilars represent a significant problem, especially for the more expensive molecules, such as adalimumab or bevacizumab. No biosimilars were available on the Slovak market in 2020 for enoxaparin, somatotropin, insulin lispro, and insulin aspart.

## Discussion

Drug policies concerning generics and their impact on reimbursement and utilization are playing an important role in releasing of valuable financial resources (Godman et al., 2010). Simple and efficient policies are required to achieve financial efficiency due to price competition among generics (Kwon and Godman, 2017). Policy tools that can increase price competition among off-patent medicines by generic substitution, along with internal and external price referencing, are evident in many countries (Kaló et al., 2021). The key target for public healthcare payers is to reinforce the allocative efficiency of healthcare costs (Inotai et al., 2017; Petykó et al., 2021). The aim of an off-patent drug policy, considering that patients have full access to the original or reference medicines prior to their patent expiry, is usually characterized by reducing health expenditures without compromising health benefits (Kaló et al., 2015).

From an economic point of view, price regulation makes sense, unless there is a presumption that fair competition will generate a market price. This is the case for a producer that holds a monopoly, or if there are few producers on the market that compete and risk a duopoly, oligopoly, or price cartel. Price regulation is not necessary if there are more competitors in the market, and public authorities minimize the risk of the cartel. High mandatory price reduction for off-patent pharmaceuticals may cause a reduction in the incentive for producers to enter the market. This is especially true if deductions are combined with strict price regulation; for example, based on so-called external referencing, the comparison of prices with other countries.

Slovakia is one of the countries with an above-average period of assessment for medicinal products following registration. This is the period from the submission of a request for the reimbursement of a generic to the granting of consent to determine the maximum price and reimbursement from public insurance. On average, this period lasts 90–120 days for generic drugs in Slovakia. For comparison, in Czechia, and Hungary, this period lasts 60 and 30 days, respectively (Golias, 2021), thus representing another disincentive for the manufacturers of generic and biosimilar medicinal products, and delaying the possibility of patients benefiting from price competition.

The obligation to reduce the prices of the first three generic medicines when entering the Slovak market (three-threshold entry) has been effective since January 1, 2018; in practice, this regulation has been applied since June 1, 2018. Mandatory price reductions, delayed decisions on reimbursements, and strict conditions for international price benchmarking, can discourage generic manufacturers from entering the market. This was also indicated by data relating to the number of submitted applications for the reimbursement of generic medicines, as published by the Ministry of Health. The number of applications for the reimbursement of generic medicines decreased from 296 in 2016 to 164 in 2019 (a 45% reduction over 3 years); there were 165 applications in 2020. Analysis based on the DDD reported that sales of generic medicines in Slovakia increased by 5.31% between 2015 and 2020. Over the same period, generic sales fell only by 0.38% in Poland, decreased by 5.56% in Hungary, and increased by 2.23% in Czechia. Financial analysis showed that sales of generic medicines in Slovakia increased by 2.77% in 2020. Over the same period, generic sales significantly fell by 13.33 in Poland and by 15.73 in Hungary, and decreased markedly by 31.06% in Czechia.

Furthermore, we also analyzed developments relating to the availability of biosimilars. It is evident that a biosimilar medicinal product can only be introduced on the pharmaceutical market with a limited discount compared to the reference medicine; this is due to high development costs compared to the discount expected from generics (Moorkens et al., 2016). However, there might still be a large total cost saving due to the high prices and significant volumes of the reference medicines (Declerck and Simoens, 2012).

Previously, researchers argued that a reduction in drug costs can be predicted not just from the launch of biosimilars but also by leveraging of the competition (Mulcahy et al., 2018). The availability of biosimilars may support competition for biological medicinal products; this may also result in the reduction of prices and increase the dynamics of the pharmaceutical market (Moorkens et al., 2019; Dutta et al., 2020). There are noticeable trends in drug policies among EU Member States concerning the price and uptake of biosimilar; this is because the potential for savings was clearly demonstrated (Vogler et al., 2021). However, the specific plan for biosimilar policies can be defined differently in countries experiencing significant resource limitations, where access to expensive reference medicines is limited (Brodszky et al., 2016; Inotai and Kaló, 2019). In countries with limited or no access to reference medicines, the key benefit of biosimilars is not related to their cost-saving potential; therefore, the target for biosimilar policies should be characterized from an investment point of view (Elek et al., 2017; Harsányi et al., 2020) by increasing patient access and by improving the number of treated patients (Inotai et al., 2018; Ferrario et al., 2020).

Following marketing authorization by the European Commission, it is the role of member states to make decisions relating to pricing and the reimbursement of biosimilars to the pharmaceutical market. Therefore, variations can be seen in the availability of biosimilars across Europe (Kawalec et al., 2017). From 2016 to the middle of 2018, not a single biosimilar became available in Slovakia; in this respect, Slovakia lagged behind other V4 and other EU countries (Tesar et al., 2020). This trend changed during the second half of 2018, in which six other biosimilars were reimbursed in Slovakia. In 2019, another 14 drugs were added; this was followed by another 11 drugs by July 2021. From July 2018 to July 2021, several drugs containing new active substances were added to the reimbursement list, including adalimumab, bevacizumab, trastuzumab, rituximab, pegfilgrastim, etanercept and teriparatide. At the end of July 2021, biosimilars for 12 molecules were available on the Slovak market. There were four other molecules with only reference (originator) medicines available (somatropin, enoxaparin sodium, insulin lispro and insulin aspart).

In terms of the DDD, sales of biosimilars in Slovakia increased by 120% between 2015 and 2020. Over the same period, biosimilars sales rose by 400% in Poland, increased by 200% in Hungary, and increased markedly by 500% in Czechia. Financial analysis showed that sales of biosimilars in Slovakia increased by 113% in 2020. Over the same period, biosimilars rose by 119 in Poland, increased by 210 in Hungary, and rose by 183% in Czechia.

With regards to reimbursement decision-making for medicines, the Ministry of Health determines its maximum price in a public pharmacy while also considering the maximum amount of reimbursement from the health insurance company, as well as the maximum amount of the insured’s co-payment. The maximum amount of the co-payment is equal to the difference between the maximum price and the maximum amount of payment. Furthermore, the Ministry classifies similar drugs (e.g., those with the same active substance or the same route of administration) in the so-called reference groups and the so-called reimbursement groups within which the same maximum reimbursement rate should apply for a standard dose of a medicinal product.

Prior to January 1, 2018, the same rules for the amount of reimbursement and co-payment applied to all medicines when the sale price of the medicine was changed. This rule for fixed co-payment was defined in the legislation as follows: “the ratio of the health insurance company’s reimbursement to the insured’s co-payment for the medicinal product (...) must remain unchanged when the selling price of the medicinal product changes ..." (Ministry of Health, 2011). With effect from January 1, 2018, the legislation was changed so that the fixed co-payment rule did not apply to a medicinal product if the additional payment of the insured was higher than 5% of the average monthly wage of an employee in the Slovak economy 2 years previously (according to the Statistical Office of the Slovak Republic, the average nominal wage was 1,013 euros in 2018, of which 5% was 50.65 euros).

The fixed co-payment rule therefore did not apply to medicinal products with a higher co-payment or to medicinal products that were in the same reference group with a medicinal product with a higher co-payment. In practice, these were usually more expensive medicines; the manufacturers of these drugs could reduce the co-payment to zero when the selling price changed. The introduction of this exemption has worsened the transparency of price competition in the sale of medicines. Upon the entry of a cheaper competitor, the manufacturer of a more expensive medicinal product was given the opportunity to reduce the selling price of their medicinal product and also the patient’s co-payment so as not to lose a competitive advantage; furthermore, there was no need to adjust the maximum state-regulated drug price in a public pharmacy. The reduction of the regulated price may be problematic for some producers due to their efforts to keep the prices from entering the international reference at a certain minimum level. However, on the basis of the reimbursement list, the manufacturer of a more expensive medicine may have a higher price and also a co-payment for the patient, but in reality, the manufacturer may sell the medicine at the same price as its competitors and without a co-payment. The actual sale price and the amount of the co-payment are not known.

According to some manufacturers, in practice, the co-payment is reimbursed to the distributor of the medicine, which means that the co-payment is not paid by the patient but by the manufacturer, for example on the basis of a credit note (Golias, 2021). This is carried out by the manufacturer of the more expensive medicine who sells the medicine to the distributor at the full official price, and then compensates for the difference between the official and the reduced price on the basis of a credit note or another form of contract. The distributor will deliver the medicine to the pharmacy at a reduced price so that the patient can receive the drug at no extra charge. Therefore, the manufacturer of the more expensive medicine has higher costs as it compensates the distributor for the price reduction, but thanks to more favorable conditions for the distributor and zero co-payment for the patient, the manufacturer is able to consolidate its competitive advantage.

Another problem with the introduction of the exception is the creation of unequal conditions for the adjustment of co-payments for more expensive and cheaper drugs, particularly medicines with high and low reimbursements from public health insurance within different reference groups. Manufacturers of more expensive medicines can gain a competitive advantage over the producers of cheaper medicines that must continue to comply with the fixed co-payment rule. This creates a paradoxical situation; from the patient’s point of view, it is more advantageous to require medicines with a higher reimbursement from public health insurance but with zero additional payment. If doctors prescribe more expensive drugs with higher reimbursement from public health insurance, then although there is a more affordable alternative with lower reimbursement from public health insurance, public resources are wasted. This exemption from the fixed co-payment may discourage the manufacturers of cheaper medicines (including generics and biosimilars) from entering the market or makes it difficult for the manufacturers to remain in the market. Furthermore, this system reduces the incentive for the manufacturers of cheaper medicines to reduce prices as it will be very difficult to change the market position of a more expensive medicine by compensating for the patient co-payment to zero.

For these reasons, the Ministry of Health prepared an amendment to Act No. 363/2011. According to this amendment, the exemption from the fixed co-payment should apply from July 1, 2020 only to those “expensive” medicines that do not have a competitor with a low co-payment. Specifically, a new point was introduced into the law; based on this new amendment, the rule of a fixed co-payment would not apply to a medicinal product whose co-payment of the insured was higher than 5% of the average monthly salary of the employee 2 years previously, if the same reference group did not include a medicine with the co-payment of the insured lower than 5% of the average monthly salary of the employee 2 years previously.

Finally, in an abbreviated legislative procedure, in June 2020, the parliament approved an amendment that stated that the fixed co-payment rule would not apply within 3 months from the end of the COVID-19 emergency situation “for a medicinal product whose insured co-payment is higher than 3% of the average monthly wage of an employee on the holding ...ˮ (Ministry of Health, 2011). In other words, the fixed co-payment exemption has been temporarily extended to medicines with a co-payment of 30.39 euros in 2020. The group of medicines for which the fixed co-payment exemption prevents transparent competition has therefore been further extended.

To intensify the use of generic and biosimilar medicines in Slovakia, it is recommended that we evaluate the importance of the existence of the three-threshold entry as well as the amount of mandatory price reductions for generic and biosimilar drugs included in the reimbursement. Price regulation is economically unjustified, particularly in the reference groups in which several competing medicines may be added. International price references may motivate manufacturers to delay the entry of a medicinal product to maintain prices at higher levels than in other countries. Therefore, we recommend considering whether the current conditions for reducing the prices of generic medicines are not too strict, especially for medicines where competition from several manufacturers and drug suppliers can be expected. It is also important that we amend the legislation so that the entry of a new package to an already included medicinal product is not considered as the entry of a new medicinal product; thus, is important with respect to the mandatory deduction from the overall price.

Based on our analysis results, our advice is to consider cancelation of the exception from the fixed co-payment of the insured. In other words, this exemption should be kept only for medicinal products for which there is no potential for competing medicinal products to enter the market. This exemption may discourage the manufacturers of cheaper medicines (including generics and biosimilars) from entering the market or make it difficult for them to remain in the market. Furthermore, this will reduce the incentive for the manufacturers of cheaper medicines to reduce prices, as it will be very difficult to change the market position of a more expensive medicine by compensating for the patient co-payment to zero.

It is also important to simplify the process and reduce the time of assessment required for generics and biosimilars after registration, and the time from submitting a request for the reimbursement of a drug to granting consent to determine the maximum price and reimbursement from public insurance. The generalization of the impacts of described reimbursement practices on pharmaceutical markets for off-patent medicines is limited. However, although we analyzed problems affecting Slovakia, we believe that other countries with low availability and penetration of off-patent medicines could also benefit from this research.

The limited length of the follow-up period and the available data allow only preliminary conclusions to be drawn on the impact of reimbursement practices on the pharmaceutical market for off-patent medicines in Slovakia.

As a consequence of the preliminary results presented herein, significant changes are needed in Slovak legislation concerning the reimbursement for off-patent medicines in Slovakia. The Slovak Ministry of Health announced for 2022 an amendment to Act No. 363/2011 that relates to the scope and conditions of payments for medicines, medical devices, and dietetic foods from public health insurance.

The prices and market shares of off-patent medicinal products vary widely across Europe (Vogler et al., 2015; Wouters et al., 2017). EU Member States are free to set the prices of medicinal products and to set up their national reimbursement requirements for medicinal products. The differences among the market shares of V4 countries for off-patent medicinal products can be explained by diverse drug policies concerning the off-patent medicinal products applied by governments in the V4. This study focused on the Slovak legislation requirements for off-patent medicinal products only. However, further research is now needed to compare differences in the legislative requirements for off-patent medicinal products among the V4; this represents a limitation in our current study.

## Data Availability

The raw data supporting the conclusion of this article will be made available by the authors, without undue reservation.
